# Providing Feedback for Learners in Virtual Visits Using a Standardized Direct Observation Assessment Form

**DOI:** 10.15694/mep.2020.000253.1

**Published:** 2020-11-11

**Authors:** Emily Sullivan, Christine Pask, Cathy MacLean, Sean Polreis

**Affiliations:** 1 University of Saskatchewan

**Keywords:** virtual care, resident supervision, assessment, competency

## Abstract

This article was migrated. The article was marked as recommended.

**Background**: The COVID-19 pandemic has created a rapid shift in primary care from in-clinic visits to virtual visits. Physicians must adapt to supervising learners in these virtual visits. Multiple factors should be considered prior to a virtual clinic day, including the type of virtual visit, the learner’s level of competence, and the patient’s characteristics and concerns. Although the approach to supervision of virtual visits may vary, direct supervision and assessment should still continue in a standardized and effective manner.

**Aim**: We have created a novel virtual visit direct observation assessment form to facilitate feedback when physicians are supervising learners in virtual care.

**Methods**: We gathered and reviewed information from the College of Family Physicians of Canada and other resources that were rapidly disseminated during the COVID-19 pandemic to create a direct observation assessment form based on evolving best practice. We conducted an internal peer-review process at our institution for the direct observation assessment form. We then distributed the form across our provincial academic sites for use.

**Results**: The authors present a standardized virtual visit direct observation assessment form for use when supervising learners. This form assesses important skills for effective patient care in a virtual setting. The criteria consist of general competencies and corresponding detailed skills.

**Conclusion**: As primary care incorporates more virtual visits, direct supervision and assessment of residents must remain a priority for academic medicine. The virtual supervision assessment form can be used as an assessment modality, a springboard for feedback, and a learning tool for residents and supervisors as they provide care in an increasingly virtual environment.

## Introduction

Virtual care has rapidly become a prominent modality of providing care in family medicine. As a result, physicians are adjusting to supervising learners during virtual visits. This type of supervision can be challenging due to a variety of reasons. How do teachers supervise their learners? Which approach for supervision in virtual care is most appropriate? What are the expectations for learners in a virtual visit?

Direct supervision and assessments are still possible and should be encouraged. Learners are less accurate when making diagnoses based on history alone compared to practicing physicians ( Tsukamoto *et al.*, 2012). The physical exam can play an important role in confirming or ruling out the differential diagnosis for learners compared to experienced physicians who may rely more on clinical gestalt. As a result, residents may seem less confident during virtual visits without a physical exam or may appear to regress in their training. Virtual visits are excellent opportunities to work through clinical reasoning, discuss selectivity, and provide formative feedback.

We have created a direct observation assessment form (see Supplementary File 1), specific to supervision of virtual visits, that can be used as a tool for resident feedback while providing virtual care. Since early March 2020 and the declaration of the COVID-19 pandemic, there has been a major shift in primary care to virtual visits. In response to this change in care delivery, the College of Family Physicians of Canada provided guidance on virtual visits and supervising residents in virtual care through webinars and guides. Using this information and reviewing other resources that were rapidly being distributed during COVID, we created a direct observation assessment form based on evolving best practices, which was then presented to our local family medicine faculty for input and feedback. Following this step, the form was further revised and sent to our residency program director for review and distribution and to the residency program committee for formal approval.

## Unique Aspects of Virtual Visits

Creating a plan, in collaboration with the learner, prior to the start of your virtual clinic can facilitate a more effective day of learning for the resident and teaching for the faculty. Make sure the learner knows how to use the technology, understands privacy concerns, and can obtain informed consent from the patient (Continuing Education and Professional Development, 2020). This is in addition to reviewing the regular outline of the day, including how patients will be seen (telephone or video), if there is direct observation, and when to review patient cases.

In order to supervise learners, it is important to understand the learner’s previous experience with virtual care ( Northern Ontario School of Medicine, 2020). The last few months may have been the first time a learner has provided care virtually via telephone or video. Ideally virtual assessments would be completed through a secure platform, such as Pexip Telemerge, rather than less secure platforms such as Skype. The experience of providing care through different formats varies for each learner, as it does for each supervisor. Platforms for virtual visits are often dictated provincially.

Virtual visits entail a unique set of privacy concerns. For example, there is a small risk of information being intercepted despite reasonable steps taken by the medical provider, such as using secure software. The patient must ensure that he or she uses a secure computer and private location. The learner should be familiar with these concerns as well as the current privacy policies in their province ( Doctors Technology Office: A GPSC Initiative, 2020).

Learners should feel comfortable obtaining informed consent from patients for virtual visits. Patients should be informed of the limitations of virtual care, such as a physician’s limited ability to do a physical examination and the possible requirement of an in-person assessment at a clinic or emergency department ( Canadian Medical Protective Association, 2020). Learners should understand these limitations in order to obtain a brief informed consent from patients. Furthermore, patients need to be informed that learners are still supervised, even when not in direct contact with the supervising physician.

## Supervision Level

The level of supervision required depends on the learner and the clinical situation ( Oandansan and Wolfrom, 2020). Similar to in-clinic care, in-coming first year residents may need more supervision than second year residents depending on their competency. As well, the clinical situation may also dictate the amount of supervision; a discussion about an advanced care directive will need closer supervision compared to a medication refill. Assess the learner’s preparedness and characteristics in the context of each clinical encounter to determine the supervision level.

Virtual visits also rely on strong communications skills. Some learners may have advanced or effective communication skills which are highlighted during phone or video visits, whereas other learners may be better with non-verbal communication, which is limited in virtual visits ( Northern Ontario School of Medicine, 2020). Furthermore, learners can benefit from observing their supervisor’s communication skills during a patient encounter, which can be difficult with virtual visits.

## Supervision Approach

The approach to supervision can occur in different ways. Supervision for learners in virtual visits can occur during the visit (synchronously) or after the visit (asynchronously). Consider the following scenarios ( Virtual Care Task Force, 2020):

• Learner and supervisor are in the same geographic location (ie. in clinic) with the patient in a different location. This can provide the opportunity for direct assessment or reviewing after the visit for both phone and video virtual visits.
• Learner, supervisor, and patient are all in separate geographic locations. The supervisor can synchronously observe the learner-patient interaction by participating in a three-way phone call or a video call; by turning off both the microphone and video camera the supervisor can optimally observe the patient-learner interaction in a “fly on the wall” scenario.
• Learner and patient in the same location with the supervisor in a different geographic location. This approach is higher risk given that it involves the least amount of oversight. As a result, the level of the learner is an important consideration if this type of supervision is chosen since there may be unchecked physical exam findings, for example.

Cases can be reviewed after each virtual visit or at the end of the day. The level of conscious competence of the learner also needs to be considered when deciding the supervision approach. Over-confident or timid learners may need more oversight despite their residency level.

Remember to review the learner’s documentation of the virtual visit. It must be clear that the virtual visit was an alternative to an in-clinic appointment, for example due to the ongoing pandemic or a geographic reason ( Oandansan *et al.*, 2020). The documentation can be used as a tool for teaching as well.

## Teaching and Evaluation

Virtual visits provide a unique opportunity for teaching. The “fly on the wall” supervision approach can provide the opportunity to observe a learner-patient interaction without being obviously “present.” Clinical decision-making and selectivity play important roles in virtual visits ( College of Family Physicians of Canada, 2020).

Clinical decision aids and algorithms can be useful during virtual care and can also be used to teach evidence-based diagnosis and selectivity in the absence of physical exam findings. For example, there are many clinical decision-making tools for the diagnosis and management of sinusitis that depend on features in the history ( Red Whale: Lifelong Learning for Primary Care, 2020). An understanding of these features, along with the red flags and when to return to clinic, can guide management during a virtual visit. This is an opportunity to use evidenced-based tools and med-ed with learners in order to guide next steps in clinical care.

Learners will need to select when it is important for a patient to have a physical examination in clinic and how the physical examination may change the management plan. Selectivity plays a role in determining even the type of virtual visit ( College of Family Physicians of Canada, 2020). Some patients may only need a phone visit, such as for reviewing results or medication refills, whereas a video visit may be more appropriate for assessing a rash or for patients who develop better rapport with face-to-face interactions. Some patients may not be appropriate for either. Some patients may simply need direction to other resources, which can be supplied by clinic staff. Other patients may need an in-clinic appointment because they do not have access to the internet or they have a medical condition that prevents them from effectively using virtual visit platforms, such as dementia or a learning disability ( Red Whale: Lifelong Learning for Primary Care, 2020). Learners need to holistically understand the patient’s context and needs to effectively choose a virtual visit format, which is a skill within itself.

Learner feedback can have different effects when given remotely. Some learners may be more comfortable with remote feedback compared to in person feedback ( Northern Ontario School of Medicine, 2020). Furthermore, some learners may receive critical feedback in a more positive way when it is given remotely.

## Direct Observation Assessment Form

The virtual supervision assessment form was created to enable supervisors to assess some of the most important skills for effectively caring for patients in a virtual setting. The criteria consist of general competencies along with some corresponding detailed skills. This comprehensive form was created using the guidance of three main documents: *Tips for Supervising Family Medicine Learners Providing Virtual Care,* *Remote consulting: a survival guide,* *and Appendix II from* *Virtual Care: recommendations for scaling up virtual medical services.* We have created a form that is practical for both supervisors and learners, and it is effective both as an assessment tool and as a springboard for giving feedback (see Supplementary File 1). Moreover, because it details a wide variety of skills pertaining to virtual visits, the form has potential benefits beyond giving residents feedback. Residents can use the form as a learning tool to prepare for an effective virtual visit, as well as to assess medical students as they begin to provide care in this environment. Furthermore, supervising physicians can hone their own virtual visit skills through the use of the form when assessing resident performance.

## Conclusion

The approach to supervision of learners during virtual visits can vary but direct assessment and feedback is still possible. The Direct Observation Assessment Form for Virtual Visits is a tool that can help facilitate feedback for learners, as well as align practitioners in their general approach to virtual visits. We wanted to share this tool for others to use, especially given that many residency programs begin in the summer and fall.

## Take Home Messages

• With COVID-19 we quickly transitioned to a world of virtual medicine and we will undoubtedly continue using this tool moving forward.
• It is important that we also prioritize supervision and direct observation of learners during virtual visits, as they require guidance and feedback to become competent in making virtual care a part of their future practice.
• A direct observation assessment form can aid in preceptor-learner communication and feedback.

## Notes On Contributors

**Emily Sullivan**, MPH, MD, CCFP is a Family Physician and faculty member in the Department of Academic Family Medicine at the University of Saskatchewan. Her research interests include preventative medicine, knowledge translation, faculty development, and maternal and infant care.

**Christine Pask**, MA, MD, CCFP is a Family Physician and faculty member in the Department of Academic Family Medicine at the University of Saskatchewan.

**Cathy MacLean**, MBA, MD, CCFP, FCFP, MCISc is the Faculty Development Director for the College of Medicine, University of Saskatchewan and a practicing family physician at West Winds Family Medicine Teaching clinic in Saskatoon, SK.

**Sean Polreis**, MEd, is the Teaching & Learning Specialist for Faculty Development at the College of Medicine, University of Saskatchewan.
