# Supplementary material for: Providing Feedback for Learners in Virtual Visits Using a Standardized Direct Observation Assessment Form
Source: MedEdPublish (2016). 2020 Nov 11;9:253. [Version 1] doi: 10.15694/mep.2020.000253.1 (PMC10697560; doi:10.15694/mep.2020.000253.1)
Supplement: Peer review history

# Reviewer report: Reviewer response for version 1

**DOI:** 10.21956/mep.20168.r31167

**Published:** 2021-04-09

**Authors:** Subha Ramani

**Affiliations:** 1 Harvard Medical School, Brigham and Women's Hospital

This review has been migrated. The reviewer awarded 4 stars out of 5 The authors have reacted well to a situation that provides challenges for all assessors of clinical trainees. The direct observation tool developed appears to include important skills required for an effective virtual encounter.I agree with some of the points raised by other reviewers:- a little more description of how the tool was developed would be useful to others- I assume the instrument is more formative than summative. In that case, formal psychometric properties could be limited to content and construct validity and interrater reliability. Even these would be needed only if the authors intend to disseminate their instrument. Otherwise, institutional educators and learners could discuss, revise and finalize. Thus, the purpose could be the deciding factor- Since this is a much needed assessment method, I agree that getting ethics approval/exemption and further research would be of value to other assessment experts.One additional thing is to be aware that not all dimensions can be observed in one televisit. This needs to be clear to assessors and those assessed. Anchoring the observation in learner goals would be most meaningful to learners.I commend the authors for designing this assessment instrument and this study.All clinical educators and assessors would find this useful.

Reviewer Expertise:

NA

No decision status is available

# Reviewer report: Reviewer response for version 1

**DOI:** 10.21956/mep.20168.r31168

**Published:** 2021-02-05

**Authors:** Ken Masters

**Affiliations:** 1 Sultan Qaboos University

This review has been migrated. The reviewer awarded 3 stars out of 5 A paper describing a standardised direct observation assessment form for providing feedback for learners in virtual visits. COVID-19 has led many healthcare providors to provide virtual patient visits, and students are largely untrained for these situations. An important part of their training is assessment, and most teachers would be unfamiliar with assessing students under these conditions. The paper provides a tool that can assist in the process.While the tool does appear to be useful, there are some areas that do need addressing:• While the form looks comprehensive, it is not enough to say that the form is based on those documents. There really does need to be some form of mapping process to show that the items on the form came from those documents. (The easiest way would be to identify each document with a number (#1,#2,#3), and then, for each item, indicate the source (and section or page, as is applicable).• Overall, the description of the form’s creation is somewhat lacking. Readers really do need to know some detail about who created it, how it was done, any kind of cross-checking, etc.• Given the urgent need for such a form, it is understandable that a rigorous evaluation could not be conducted. In spite of this, however, a small pilot of four or five supervisors and students willing to give their input could have been performed with minimal ethics clearance. This really should have been performed and documented. Smaller issue:• In the text, where the three documents are listed, they should be properly cited in the same way as the other References. • In the references, Oandansan, I. and Wolfrom is an online video, but no link is given. Please supply the link.I look forward to Version 2 of the paper in which these issues are addressed.

Reviewer Expertise:

NA

No decision status is available

# Reviewer report: Reviewer response for version 1

**DOI:** 10.21956/mep.20168.r31169

**Published:** 2020-11-14

**Authors:** Mary Higgins

**Affiliations:** 1 University College Dublin, National Maternity Hospital

This review has been migrated. The reviewer awarded 4 stars out of 5 Thank you for this very interesting paper, hugely useful in our new world. May I ask have you any plans to validate the tool - to get feedback from users, to check face and content validity, to assess practicality of use?

Reviewer Expertise:

NA

No decision status is available
